# Intravenous flurbiprofen for post-thymectomy pain relief in patients with myasthenia gravis

**DOI:** 10.1186/1749-8090-7-98

**Published:** 2012-09-29

**Authors:** Chunhua Su, Yihua Su, Chiu-Wen Chou, Weibing Liu, Jianyong Zou, Honghe Luo, Zhenguang Chen

**Affiliations:** 1 Department of Thoracic Surgery Lung Cancer Research Center, The First Affiliated Hospital, Sun Yat-sen University, No. 58, Zhongshan Road 2, Guangzhou, Guangdong, 510080, People’s Republic of China; 2 Department of Ophthalmology, The First Affiliated Hospital, Sun Yat-sen University, Guangzhou, Guangdong, 510080, People’s Republic of China; 3 Department of Anesthesiology, The First Affiliated Hospital, Sun Yat-sen University, Guangzhou, Guangdong, 510080, People’s Republic of China; 4 Department of Neurology, The First Affiliated Hospital, Sun Yat-sen University, Guangzhou, Guangdong, 510080, People’s Republic of China; 5 Department of Cardiothoracic Surgery in Huangpu Division, The First Affiliated Hospital, Sun Yat-sen University, No. 58, Zhongshan Road 2, Guangzhou, Guangdong, 510080, People’s Republic of China

**Keywords:** Myasthenia gravis, Thymectomy, Postoperative care

## Abstract

**Background:**

Post-thymectomy pain in myasthenia gravis (MG) patients can inhibit breathing and coughing. Inappropriate usage of analgesics may exacerbate respiratory inhibition and even cause myasthenic crisis. Flurbiprofen is a non-steroidal anti-inflammatory drug (NSAID) that is commonly used to control moderate postoperative pain and is not associated with respiratory inhibition. We hypothesized that flurbiprofen may provide post-thymectomy pain relief without increasing the risk of complications in MG patients.

**Methods:**

Two hundred MG patients underwent extended thymectomy from March 2006 to December 2010 and were randomly allocated to a flurbiprofen group (110 patients, 50 mg intravenous flurbiprofen axetil) or a control group (90 patients, 100 mg intramuscular tramadol) as postoperative analgesia. Visual analog scale (VAS) pain score, heart rate, blood pressure, respiratory rate, pulse oximetry (SpO_2_), and adverse effects were recorded before and up to 24 h after drug administration.

**Results:**

There were no significant differences in the preoperative clinical characteristics of the flurbiprofen and control (tramadol) groups. Both flurbiprofen and tramadol significantly alleviated post-thymectomy pain (*p* < 0.05 for both), but patients in flurbiprofen group had significantly lower VAS pain scores at 0.5 h, 2 h, 4 h, and 8 h after surgery (*p* < 0.05 for all times). There were no significant post-thymectomy changes of heart rate, respiratory rate, mean arterial blood pressure, or SpO_2_ in either group at all time points.

**Conclusions:**

Post-thymectomy intravenous administration of flurbiprofen axetil provides safe and effective analgesia for MG patients.

## Background

Myasthenia gravis (MG) is an autoimmune disorder characterized by progressive muscle fatigue due to alterations of the postsynaptic neuromuscular junction. Most patients also experience abnormalities of the thymus gland, such as thymoma or benign thymus hyperplasia [1,2]. Thymectomy in the early stages of MG often improves the overall prognosis [3,4]. The extended transsternal thymectomy is believed as a routine and complete surgical dissection procedure [5-9], and video-assisted thoracic surgery (VATS) thymectomy and robotic-aided video-assisted thoracoscopic thymectomy are continually being developed [10,11]. Based on series of 5-year follow-up data, combined cervical and bilateral videoscopic thymectomy and the combined transcervical–transsternal thymectomy have 51 and 50%, respectively, remission rates at that interval. Similarly, extended transsternal thymectomy has 40% remission rate and a high percentage of patients with ocular or mild preoperative MG manifestations [12-14]. It suggests that at the extended or combined transcervical transsternal thymectomy have the better remission rates and the outcomes of different minimally invasive approach are still in the investigative phase. Considering that the extended transsternal thymectomy is conventional until now, the management of postoperative pain is often a significant problem in MG patients who have undergone thymectomy via median sternotomy incision [15,16]. Surgical pain following thymectomy may disrupt breathing and inhibit coughing, and thus may hamper patient recovery and even lead to myasthenic crisis [16,17].

The choice of analgesic agent and route of administration are important because post-thymectomy pain may compromise pulmonary function, and hypersensitivity to an analgesic agent may induce respiratory depression. Previous research reported good results with high thoracic (C_7_T_1-3_) epidural combinations of bupivacaine and morphine in a small number of patients, but the paucity of such cases and the technical difficulties associated with administration of thoracic epidural analgesia have prevented acceptance of this technique [18]. However, there are few established analgesics for treatment of post-thymectomy pain in these patients. Traditional analgesics, such as tramadol, may cause severe nausea and vomiting, and inhibit respiration [19,20]. All analgesics must be used with care in post-thymectomy patients with MG [21].

Non-steroidal anti-inflammatory drugs (NSAIDs) also provide relief from pain after different types of surgery [22,23]. Flurbiprofen is an NSAID that can safely and effectively control moderate postoperative pain [23,24]. A recent Cochrane review reported that oral flurbiprofen at a dose of 50 mg or 100 mg provided at least 50% pain relief in 65% to 70% of postoperative patients and only had a weak inhibitory effect on respiration [25]. Systemic flurbiprofen given as treatment for postoperative pain following thoracotomy reduces serum levels of IL-6 and CRP and appears to contribute to the attenuation of the postoperative inflammatory response and prevent postoperative pain [26]. However, the effect of intravenous flurbiprofen on post-thymectomy pain relief in patients with MG has not been established.

In the present study, we investigated the effect of intravenous flurbiprofen on relief from post-thymectomy pain in patients with MG.

## Methods

### Patient data

MG patients who underwent extended thymectomy in the Department of Thoracic Surgery of the First Affiliated Hospital of Sun Yat-sen University (Guangzhou, China) from March 2007 to December 2010 were enrolled. Clinical information was obtained by review of the preoperative and perioperative medical records. MG was diagnosed based on patient history, clinical symptoms, chest computed tomography, positive response to the edrophonium chloride test, and positive findings by single-fiber electromyography and was staged by criteria of the MG Foundation of America [27,28]. Anticholinesterase agents were administered by neurology consultants, and none of the patients received immunosuppressive agents preoperatively. All patients underwent extended thymectomy via median sternotomy followed by standard wound closure, as described previously [29]. Patients were randomly assigned to the flurbiprofen group (50 mg intravenous flurbiprofen axetil diluted in 0.9% sodium chloride, completed within 1 h) or to the control group (100 mg intramuscular tramadol) for postoperative analgesia. The duration of surgery, time of chest drainage, MGFA stage at 72 h after operation, presence of myasthenia crisis, and adverse reactions were recorded. The hospital ethics committee approved the study protocol (No. 2012–038).

### Evaluation of the postoperative pain

A self-reported visual analog scale (VAS) was used to evaluate postoperative pain, in which 0 indicated “no pain”, 1 to 3 indicated “mild pain”, 4 to 6 indicated “moderate pain”, and 7 to 10 indicated “severe pain”. The severity of nausea and vomiting was recorded with a four-point scale in which 0 indicated “no nausea or vomiting”, 1 indicated “no nausea at rest or nausea with mild vomiting on exertion”, 2 indicated “intermittent nausea at rest or nausea with moderate vomiting on exertion, and 3 indicated “persistent nausea at rest or severe nausea with severe vomiting on exertion”. Mean arterial blood pressure, heart rate, respiratory rate, and SpO_2_, were recorded before surgery and at 0.5 h, 2 h, 4 h, 8 h, and 24 h after administration of analgesics. A myasthenic crisis was defined as respiratory failure caused by respiratory muscle weakness. Adverse events, including gastrointestinal bleeding, ulcers, dizziness, lethargy, flushing, itching, urinary retention, constipation, respiratory inhibition, abnormal bleeding, were also recorded. Nausea and vomiting were treated with 10 mg of intramuscular metoclopramide, and headache was treated with 60 mg of intramuscular rotundine.

### Statistical analysis

All statistical calculations were performed using SPSS ver. 14.0 (Chicago, Illinois, USA). All data are given as means ± standard deviations. The chi-square test and the Mann–Whitney test were used for comparisons, as appropriate. For all tests, a two-sided *p*-value less than 0.05 was considered significant. Hazard ratios (HR) and 95% confidence intervals (CIs) were calculated to assess the significance of differences.

## Results

### Basic clinical information

Our study cohort consisted of 200 patients with non-thymomatous MG who were treated by extended thymectomy (Table 1). There were 98 males and 102 females, the mean age was 30.7 ± 12.3 years (range, 15–69 years; median, 27.2 years), and the mean duration of symptoms was 1.72 ± 0.21 years (range, 0.5-23 years; median, 1 year). A total of 84 patients were MGFA stage I, 74 were stage IIa, 32 were stage IIb, and 10 were stage IIIa.

**Table 1:** **Clinical and demographic characteristics of the flurbiprofen and control (tramadol) groups **

**Characteristic**	**Flurbiprofen**	**Control (tramadol)**	*p*value*
*n*	110	90	
Male/female, *n* (%)	59 (53.6)/51 (46.4)	43 (47.8)/47 (52.2)	0.504
Age at surgery, years	31.1 ± 11.8	30.3 ± 13.1	0.691
Mean disease duration, years	1.5 ± 0.8	1.8 ± 0.9	0.401
With/without medulla symptoms, *n* (%)	17 (15.5)/93 (84.5)	15 (16.7)/75 (83.3)	0.816
MGFA stage, *n* (%)			0.954
I	46 (41.8)	38 (42.2)	
IIa	41 (37.3)	33 (36.7)	
IIb	17 (15.5)	15 (16.7)	
IIIa	6 (5.45)	4 (4.44)	
Time of operation (min)	111.8 ± 25.4	116.4 ± 24.0	0.843
Time of chest drainage (d)	2.7 ± 0.5	2.4 ± 0.8	0.927
Myasthenia crisis	4 (3.6)	3 (3.3)	0.911
MGFA stage at 72 h after operation, *n* (%)			0.931
I	47 (42.7)	39 (43.3)	
IIa	42 (38.2)	33 (36.7)	
IIb	15 (13.6)	14 (15.6)	
IIIa	6 (5.45)	4 (4.44)	
Adverse reactions			
Nausea and vomiting	14 (12.7)	26 (28.9)	0.021
Headache	5 (4.5)	9 (10.0)	0.162
Facial flushing	3 (2.7)	4 (4.4)	0.526

Values are given as median ± SD or (percentage). MGFA, Myasthenia Gravis Foundation Association.

* *p*-values were calculated by the *χ*^2^ test.

A total of 110 patients were randomized to the flurbiprofen group and 90 patients to the control (tramadol) group. After surgery, seven cases of myasthenic crisis were recorded, with four cases in the flurbiprofen group (two cases with stage IIb and two cases with stage IIIa), and three cases in the control group (one case with stage IIb and two cases with stage IIIa). Nausea and vomiting were significantly more common in the control group (28.9% *vs.* 12.7%, *p* = 0.021), but there were no other significant differences between the flurbiprofen and control groups.

### Assessment of postoperative pain

**Figure 1:**
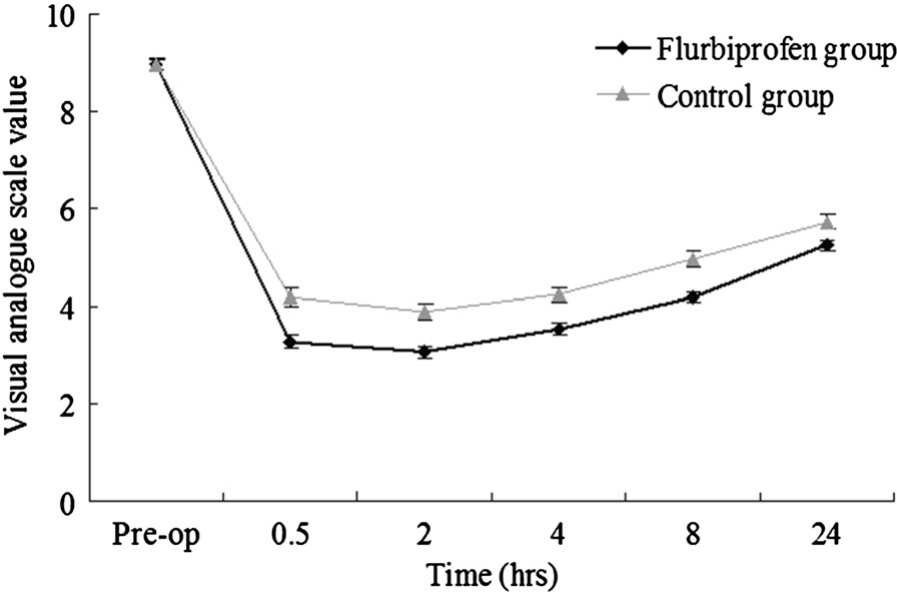
**Comparison of visual analogpain scores in theflurbiprofen and control groups.** An asterisk indicates a significant difference (*p* < 0.05).

**Figure 2:**
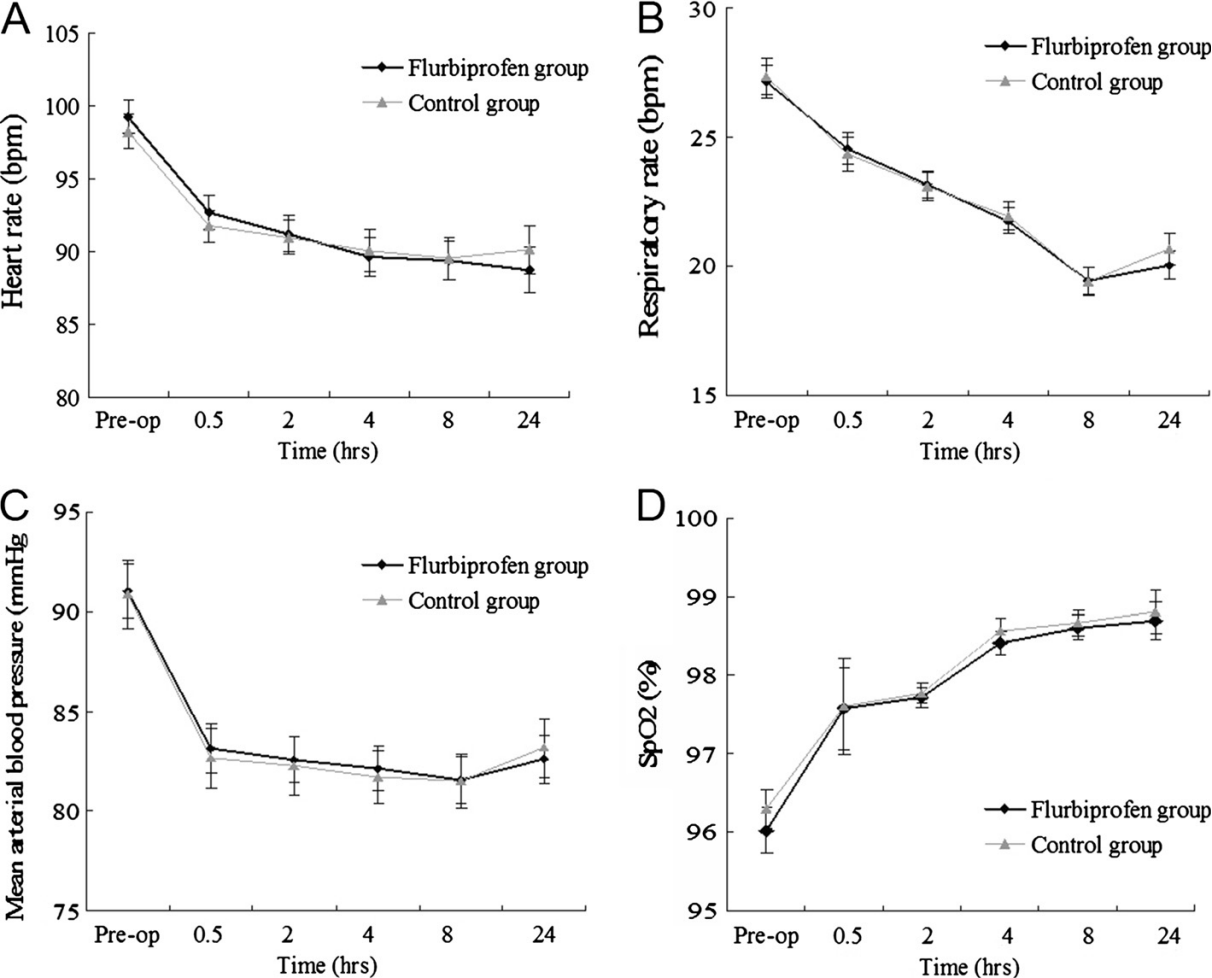
**A, Comparison of heartbeat variation in theflurbiprofen and control groups;B, Comparison of respiratoryrate variation in theflurbiprofen and control groups;C, Comparison of meanarterial blood pressure variationin the flurbiprofen andcontrol groups; D, Comparisonof variation in bloodoxygen saturation in theflurbiprofen and control groups.**

Before analgesic administration, patients in both groups complained of wound pain, difficulty with deep breathing, and coughing, and all patients had VAS pain scores of 7 or higher. Our results indicate that both drugs significantly alleviated postoperative pain and that the mean VAS pain score was significantly lower in the flurbiprofen group at 0.5 h, 2 h, 4 h, and 8 h after surgery (*p* < 0.05) (Figure 1). There were no differences between the two groups in heart rate (Figure 2A), respiratory rate (Figure 2B), mean arterial blood pressure (Figure 2C), or oxygen saturation (Figure 2D) for up to 24 h after surgery.

### Adverse reactions

Nausea and vomiting were significantly more common in the control group than in the flurbiprofen group (12.7% *vs.* 28.9%, *p* = 0.021, Table 1). At 30 min after initiation of analgesia, there were no adverse reactions in either group. In the flurbiprofen group, there 14 cases of nausea and vomiting at 2 h, (6 cases with scores of 3, 8 cases with scores of 2), 9 cases of nausea and vomiting at 4 h, and 5 cases of nausea and vomiting at 8 h. In the control group, there were 26 cases of nausea and vomiting at 2 h (12 cases with scores of 3, 14 cases with scores of 2), and 16 cases of nausea and vomiting at 4 h and 8 h.

At 2 h after drug injection, five cases in flurbiprofen group and nine cases in control group had headaches, but all of these patients experienced remission at 4 h. Remarkably, three cases in the flurbiprofen group and four cases in the control (tramadol) group had facial flushing at 2 h after drug injection, and these symptoms remained for 24 h in all patients.

## Discussion

Extended thymectomy is recognized worldwide as an important technique to manage myasthenia gravis, and median sternotomy is the major surgical method [1,3,27]. The injury caused by this surgery is considerable, because the pleura are often damaged during resection of anterior mediastinal fat tissue. Surgery may lead to hemothorax or pleural effusion that can affect pulmonary re-expansion. Coughing, sputum expulsion, and initiation of deep breathing are important measures that promote lung re-expansion and reduce the risk of pulmonary infection. The surgical pain of such patients typically has medium-to-high intensity, and may inhibit breathing, coughing, and sputum expulsion, thereby disrupting postoperative recovery, increasing the risk of pulmonary atelectasis and infection, and even induce myasthenic crisis [30]. Our previous study of MG patients demonstrated that the serum concentration of immunoglobulin G was high after thymectomy [31]. Another report showed that the levels of serum CRP, IL-6, and TNF-α were elevated after thoracotomy [26]. Together, these findings indicate significant inflammation after thymectomy, suggesting that an anti-inflammatory agent such as flurbiprofen may be effective in reducing this inflammation.

Common analgesics, such as tramadol and adolens, are often not recommended for MG patients because they can depress respiration and are associated adverse effects (nausea, vomiting, irritability) that can increase the risk of a myasthenic crisis or worsen postoperative conditions. Therefore, an effective analgesic is needed that allows pain-free recovery from thymectomy but does not inhibit the respiratory muscles.

Flurbiprofen axetil (2-[3-fluoro-4-biphenyl] propanoic acid) is an NSAID of the propionic acid class and tablets are approved for relief of pain from rheumatoid arthritis and osteoarthritis [32]. Intravenous flurbiprofen axetil is provided as 50 mg ampoules and selectively accumulates at the surgical incision and inflammation sites after administration. Flurbiprofen axetil is mostly metabolized into flurbiprofen by hydrolysis and its analgesic effect is believed to result from the reversible inhibition of cyclooxygenase and peripheral inhibition of prostaglandin synthesis. Analgesia has been reported to last for up to 24 h after intravenous administration [33]. During postoperative pain management with flurbiprofen, patients must be monitored for side effects, especially gastrointestinal effects, as with all NSAIDs [2,34]. Previous studies have reported that use of flurbiprofen for postoperative analgesia in anesthesia, general surgery, obstetrics and gynecology, and orthopedics was effective and safe, had little effect on respiration, and prolonged the duration of analgesia [35-38]. However, there have been no reports on the use of flurbiprofen following thoracic surgery or in patients with MG following extended thymectomy.

Our results indicate that intravenous flurbiprofen axetil can effectively reduce post-thymectomy pain without disruption of respiratory rate, heart rate, or blood pressure. The analgesic effect lasted for up to 24 h for most patients. There was no significant difference in the incidence of myasthenic crisis in the flurbiprofen and control groups and there were fewer adverse effects in the flurbiprofen group. The most common adverse effects following flurbiprofen axetil were headache, vomiting, and flushing. Mild symptoms resolved by themselves within 3 days, and there were more cases of adverse reactions in the control group. Slow intravenous administration of flurbiprofen allows immediate withdrawal if respiratory inhibition occurs and provides an extended duration of analgesia.

This study confirms that flurbiprofen axetil is a safe and effective analgesic for post-thymectomy patients with MG and that it is associated with a lower incidence of adverse reactions than tramadol. Thus, slow intravenous administration of flurbiprofen axetil is safe for clinical use and is not associated with respiratory inhibition.

## Competing interests

The authors declare no competing interests.

## Authors’ contributions

CS and ZC designed the study, collected data, and drafted the manuscript. CC performed the statistical analysis and drafted the manuscript. YS, YL, JZ, BZ participated in data collection. HL participated in the study design and coordination and helped to draft the manuscript. All authors read and approved the final manuscript.
